# Transcriptome Profile Analysis on Ovarian Tissues of Autotetraploid Fish and Diploid Red Crucian Carp

**DOI:** 10.3389/fgene.2019.00208

**Published:** 2019-03-19

**Authors:** Yude Wang, Minghe Zhang, Qinbo Qin, Yajun Peng, Xu Huang, Chongqing Wang, Liu Cao, Wuhui Li, Min Tao, Chun Zhang, Shaojun Liu

**Affiliations:** State Key Laboratory of Developmental Biology of Freshwater Fish, College of Life Sciences, Hunan Normal University, Changsha, China

**Keywords:** red crucian carp, autotetraploid fish, ovarian tissues, egg size, transcriptome

## Abstract

Polyploidization can significantly alter the size of animal gametes. Autotetraploid fish (RRRR, 4nRR = 200) (4nRR) possessing four sets of chromosomes were derived from whole-genome duplication in red crucian carp (RR, 2*n* = 100) (RCC). The diploid eggs of the 4nRR fish were significantly larger than the eggs of RCC. To explore the differences between the ovaries of these two ploidies of fishes at the molecular level, we compared the ovary transcriptome profiles of 4nRR fish and RCC and identified differentially expressed genes (DEGs). A total of 19,015 unigenes were differentially expressed between 4nRR fish and RCC, including 12,591 upregulated and 6,424 downregulated unigenes in 4nRR fish. Functional analyses revealed that eight genes (*CDKL1*, *AHCY*, *ARHGEF3*, *TGFβ*, *WNT11*, *CYP27A*, *GDF7*, and *CKB*) were involved in the regulation of cell proliferation, cell division, gene transcription, ovary development and energy metabolism, suggesting that these eight genes were related to egg size in 4nRR fish and RCC. We validated the expression levels of these eight DEGs in 4nRR fish and RCC using quantitative PCR. The study results provided insights into the regulatory mechanisms underlying the differences in crucian carp egg sizes.

## Introduction

Polyploidy is a very common phenomenon. In vertebrate evolution, polyploidy is considered to have led to the evolution of more complex forms of life by providing the opportunity for new functions to evolve (Ohno, 1970; Epstein, 1971). Polyploidy, including allopolyploidy and autopolyploidy, is both widespread and evolutionarily important (Van de Peer et al., 2017). Allopolyploids contain genomes from distinct taxa, while autopolyploids are formed by genomes from the same species (Van Drunen and Husband, 2018).

Phenotypic changes induced by chromosome duplications have been reported since the early 20^th^ century (Stebbins, 1947). A well-known effect of polyploidy in plants and animals is cell enlargement (Knight and Beaulieu, 2008), but less evident effects can also occur (Maherali et al., 2009). In plants, for example, polyploidy often modifies physiological traits such as transpiration, and rates of photosynthesis or growth (Levin, 2002). Following such changes in physiology, shifts in ecological tolerance have been demonstrated for some taxa (Levin, 2002). Polyploidy can also induce phenotypic modifications in reproductive traits, but surprisingly, these effects have received less attention. Sometimes, polyploids have reproductive organs that are larger than those of their diploid counterparts (Robertson et al., 2011). Following their instantaneous multiplication in DNA content, polyploids can experience processes that either expand or shrink their genomes (Leitch et al., 2008). This increase in DNA has great potential to induce phenotypic variation (Chen, 2007). The relationships between genome size and phenotypic traits have been discussed in comparative studies at a broad phylogenetic levels (Muenzbergova, 2009), but few studies have analyzed how and whether genome size or polyploidy can modify phenotypic traits at the microevolutionary scale (Lavergne et al., 2009). In fish, polyploidization can obviously alter egg size. For example, allotetraploid hybrids of red crucian carp **×** common carp can produce diploid eggs that are obviously larger than those of their parents (Liu et al., 2004, 2007). Forés et al. (1990) studied egg activity in *Scophthalmus maximus* and found that these eggs reached a higher fertilization ratio when the diameter of the egg was 0.9–1.1 mm, but when the diameter was 1.1–1.2 mm, the fertilization ratio was lower. Thus, egg diameter is an important parameter that can reflect the positives or negatives of egg mass. Autotetraploid fish (4nRR) (Qin et al., 2014) derived from genome duplication in RCC. Autopolyploids often differ ecologically and phenotypically from their low ploidy parents (Husband et al., 2016), but because studies are commonly performed on long-established cytotypes (Comai, 2005). It is unclear whether differences are due to instantaneous changes associated with the whole-genome duplication (WGD) event or divergences through selection after the fact (Weiss-Schneeweiss et al., 2013). In this process, we found that egg diameters of 4nRR fish are larger than those of RCC. Meanwhile, through self-crossing RCC and 4nRR fish, we found that the fertilization ratio of RCC (96.70%) was higher than that of 4nRR fish (65.36%). In livestock and wildlife, egg quality is affected by a number of factors and is highly variable, including egg size (Chapman et al., 2014). Egg size plays an important role in the heredity and reproduction of fish.

In this study, we examined the transcriptomes of mature ovarian tissues from 4nRR fish and RCC using RNA-seq. The purposes of this research were to expand the genetic resources available for crucian carp, analyze differentially expressed genes (DEGs) between 4nRR fish and RCC and identify genes related to egg diameter. Overall, our results were valuable for understanding valuable genomic information and the molecular mechanism of ovarian development in 4nRR fish and RCC. In addition, this study helped establish a foundation for polyploid evolution and molecular breeding in crucian carp and other closely related species.

## Materials and Methods

### Ethics Statement

Fish researchers were certified under a professional training course for laboratory animal practitioners held by the Institute of Experimental Animals, Hunan Province, China (Certificate No. 4263). All fish were euthanized using 2-phenoxyethanol (Sigma, United States) before dissection. This study was carried out in accordance with the recommendations of the Administration of Affairs Concerning Experimental Animals for the Science and Technology Bureau of China. The protocol was approved by the Administration of Affairs Concerning Experimental Animals for the Science and Technology Bureau of China.

### Sample Collection and Preparation

One-year old female 4nRR fish and RCC were obtained from the State Key Laboratory of Developmental Biology of Freshwater Fish, Hunan Normal University, China. The ploidy status of the 4nRR fish and RCC was tested by flow cytometry as described by Qin et al. (2014). Three one-year-old mature female 4nRR fish and RCC were chosen. Ovarian tissues were removed from the 4nRR fish and RCC after euthanasia using 2-phenoxyethanol (Sigma, United States). In this experiment, 4nRR fish were used as treatment group, while RCC was used as a control group. The ovarian tissues of the 4nRR fish and RCC were then divided into three parts; the first part was used to measure egg diameters to test differences in egg size between 4nRR fish and RCC using multiple-contingency-table analyses (Sokal and Rohlf, 1981); the second part was fixed in 4% paraformaldehyde solution for histological observation as described in Cao and Wang (2009); the third part was promptly frozen in liquid nitrogen, stored at –80°C, and then used for RNA-Seq and Real-time Quantitative PCR Detecting System (qPCR) analysis. Total RNA was extracted from 4nRR fish and RCC ovarian tissues using a Total RNA Kit II (TaKaRa, China) according to the instructions of the manufacturer. For each ploidy. Each amounts of RNA from three 4nRR fish and three RCC were pooled to offer templates for construction of the RNA-Seq library (Supplementary Figure 1).

### Measurement of the Size of the Eggs and the Histology Observation of Ovary

Ten female 4nRR fish and ten female RCC were sorted into two groups producing “high-quality” or “low-quality” eggs as described by Chapman et al. (2014). The diameters of 167 4nRR fish eggs and 167 RCC eggs were measured by a Vernier caliper. We used analyses of variance (ANOVA) (Osterlind et al., 2001) and multiple comparison tests (LSD method) (Williams and Abdi, 2010) to test for differences in egg size between 4nRR fish and RCC using SPSS Statistics 21.0. The values of the independent variables were expressed as the mean ± SD. The gonads of 4nRR fish and RCC were fixed in Bouin’s solution for the preparation of tissue sections. The paraffin-embedded sections were cut and stained with hematoxylin and eosin. Gonadal structure was observed with a light microscope and photographed with a Pixera Pro 600ES.

### RNA Sequencing Library Construction and Illumina Sequencing

The cDNA library was constructed using high quality RNA. Poly (A) was separated using oligo-dT beads (Qiagen, Dusseldorf, Germany). The fragmentation buffer was added to break all the mRNA into short fragments. Random hexamer-primed reverse transcription was used for first-strand cDNA synthesis. The second cDNA strand synthesis was subsequently performed using DNA polymerase I and endonuclease. The quick PCR extraction kit was used to purify the cDNA fragments. These purified cDNA fragments were rinsed with EB buffer for end reparation Poly (A) addition and then ligated to sequencing barcodes. The fragments with a size suitable for sequencing criteria were isolated from the gels and enriched by PCR amplification to construct the final cDNA library. The cDNA library was sequenced on the Illumina sequencing platform (Illumina Hiseq^TM^2500) using paired-end technology in a single run, by Novogene Technologies (Beijing, China). The Illumina GA processing pipeline was used to analyze the images and for base calling.

### *De novo* Assembly and Functional Annotation

Raw reads were filtered using Fastqc software (Babraham Bioinformatics) (Davis et al., 2013) to obtain paired-end clean reads. All clean reads were used for assembly using Trinity software (Grabherr et al., 2011) with the following parameters: (1) minimum assembled contig length to report = 100bp; (2) maximum length expected between fragment pairs = 250 bp; and (3) count for K-mers to be assembled by Inchworm = 25. After assembly, contigs longer than 200 bp were used for analysis. The contigs were connected to obtain sequences that could not be extended further at either end, and the sequences of the unigenes were generated. The unigenes were further spliced and assembled to acquire maximum length non-redundant unigenes using TGICL clustering software (J. Craig Venter Institute, Rockville, MD, United States). Finally, Blastx was used to compare the unigenes base on *E*-value < 10^-5^ (Altschul et al., 1997) with the non-redundant protein (Nr), SwissProt, Kyoto Encyclopedia of Genes and Genomes (KEGG) and Clusters of Orthologous Group (COG) databases (*E*-value < 10^-3^). Gene ontology (GO) annotation of the unigenes was completed using Blast2GO based on the results from the NCBI Nr database annotation. Blastn was used to align the unigenes to the Nr database and search for proteins with the highest sequence similarity to the given unigenes, accompanied by their protein functional annotations. A heat map which grouped genes according to FPKM values was generated in Cluster3.0 (De Hoon et al., 2004).

### Identification of Differentially Expressed Genes (DEGs)

The mapped reads were normalized according to the FPKM for each unigene between the 4nRR and RCC fish, which was beneficial for comparing unigene expressions (McCarthy and Smyth, 2009) of 4nRR and RCC fish. The DEGs were identified by the DEGseq package (Wang et al., 2009) by applying the MA-plot-based method with a random sampling model. DEGs between 4nRR and RCC fish were selected based on the following filter criteria: (1) false discovery (FDR) < 0.05; and (2) |log_2_(foldchange)| > 1 (Storey and Tibshirani, 2003; Lv et al., 2013).

### Validation of RNA-Seq Results by qPCR

To verify the reliability of the RNA-seq results, eight DEGs (*CDKL1*, *CKB*, *AHCY*, *ARHGEF3*, *TGFβ*, *SCP1*, *WNT11* and *CYP27A*) involved in the development of ovarian tissues were selected for validation using quantitative real-time PCR (qPCR) on a Prism 7500 Sequence Detection System (Applied Biosystems, United States) with a miScript SYBR Green PCR Kit (Qiagen, Germany). The reaction mixture (10 μL) comprised 2.5 μL cDNA (1:3 dilution), 5 μL SYBR Premix Ex TaqTMII (TaKaRa), 0.5 μL specific forward primer, 0.5 μL reversal primer, and 1.5 μL water. Real-time PCR was performed on biological replicates in triplicate. The amplification conditions were as follows: (1) 50°C for 5 min, (2) 95°C for 10 min and (3) 40 cycles at 95°C for 15 s, followed by 60°C for 45 s. The average threshold cycle (Ct) was calculated for 4nRR fish and RCC using the 2^-ΔΔCt^ method (Pfaffl, 2001) and normalized to that of β-actin. Finally, a melting curve analysis was completed to validate the specific generation of the expected products.

## Results

### Comparison of Egg Size

**FIGURE 1:**
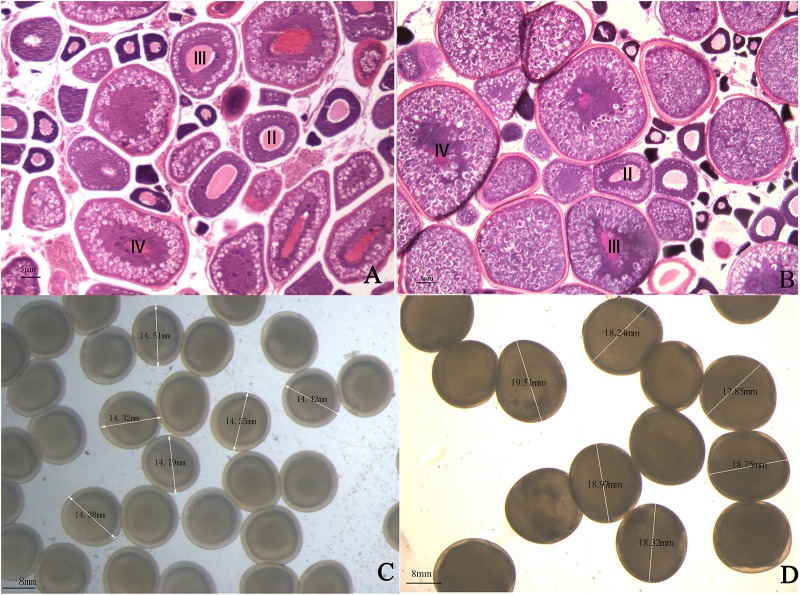
Gonadal structure of ovarian tissues in **(A)** red crucian carp and **(B)** autotetraploid crucian carp. The eggs produced by **(C)** red crucian carp and **(D)** autotetraploid fish.

One-year-old 4nRR and RCC fish were used in this research. The ovaries of one-year 4nRR and RCC fish developed well and contained II, III, and IV oocytes. Furthermore, large numbers of eggs were stripped from one-year-old 4nRR fish and RCC, respectively. The results showed that 4nRR and RCC fish had reached sexual maturity by one year of age (Figure 1A,B). The average egg diameters of the RCC and 4nRR fish were 13.67 and 17.71 mm, respectively (Table 1). Eggs from 4nRR fish were significantly larger than those from RCC fish (Figure 1C,D) (*t* = -33.370, *p* < 0.05).

**Table 1:** Comparison of mature eggs diameters between RCC and 4nRR fish (Each fish is randomly selected 10).

Ploidy	Egg Numbers	Egg Diameters (mm)
2n	167	13.67 ± 0.03^a^
4n	167	17.71 ± 0.08^b^


*Means in same column with different superscripts were very significantly different (*t* = –33.370, *p* < 0.01).*

### Sequencing, *de novo* Assembly and Functional Annotation

RNA-seq (Feng et al., 2012) was conducted on 4nRR and RCC fish ovarian tissue. A total of 118.1 million 150 bp paired-end reads were generated. After removing low-quality reads and short read sequences, a total of 108.1 million clean reads (91.54%) were obtained (Supplementary Table 1), and these reads were used for the following analyses. Ovarian tissues from RCC and 4nRR fish were used to generate 212,573 transcripts and 149,851 unigenes. The N50 values of the transcripts and unigenes were 1,525 and 996 bp, respectively. A summary of the assembly data was shown in Supplementary Table 2. The length distributions of the transcripts and unigenes were shown in Supplementary Figure 2. Approximately 91.9% of the unigenes (149,861) were annotated by Blastx and Blastn against seven databases (GO, KO, KOG, NR, NT, PFAM, and SwissProt) with a threshold of 10^-5^ (Altschul et al., 1997; Lv et al., 2013). Among these unigenes, 38,140, 26,510, 21,296, 51,507, 135,474, 36,236, and 40,008 were identified in the GO, KO, KOG, NR, NT, PFAM and SwissProt databases, respectively (Supplementary Figure 3). Clean RNA sequencing reads were deposited in the NCBI Sequence Read Archive (SRA) under accession numbers SAMN07418623 and SAMN07418624^1^.

### The Differentially Expressed Genes Between the Two Kinds of Crucian Carp

A total of 19,015 unigenes were differentially expressed between the RCC and 4nRR fish. In total, 12,591 unigenes were upregulated in 4nRR fish, while 6,424 unigenes were downregulated in 4nRR fish compared with RCC. Some upregulated genes in 4nRR fish, such as *vitellogenin* (*Vtg*), *Meiotic nuclear division 5 homolog B* (*Md5b*), *Mediator of RNA polymerase II transcription subunit 25* (*Mpts*), *Transcription factor TFIIIB component* (*Tfc*), *Cell division cycle-associated protein 3*(*Cdc3*), *S-phase kinase-associated protein* (*Skp1*), *Bcl-2-related ovarian killer protein homolog A* (*Bokp*), *Ovarian cystatin* (*Oct*), *Dynein regulatory complex protein 1* (*Drc1*) and *Cyclin-dependent kinase-like 1* (*CDKL1*) (Table 2), were mainly involved in the regulation of cell proliferation and cell division, gene transcription, ovary development and energy metabolism, showing that these genes might be related to egg diameter in crucian carp.

**Table 2:** Summary of the 10 DEGs related to egg diameter.

Gene id	Gene name	Log_2_ ratio (4n/2n)	FDR^∗^
c9025_g1	Vitellogenin	0.74981	0.011572
c95832_g2	Meiotic nuclear division 5 homolog B	1.2554	3.43 × 10^-6^
c95731_g1	Mediator of RNA polymerase II transcription subunit 25	0.68799	0.016038
c85064_g3	Transcription factor TFIIIB component	2.5585	0.031803
c93446_g1	Cell division cycle-associated protein 3	0.9603	0.029923
c87372_g1	S-phase kinase-associated protein1	4.0071	0.0001137
c77438_g1	Bcl-2-related ovarian killer protein homolog A	4.9119	1.14 × 10^-7^
c88390_g4	Ovarian cystatin	10.811	2.80 × 10^-34^
c85100_g1	G2/M phase-specific E3 ubiquitin-protein ligase	1.6731	2.35 × 10^-8^
c85551_g1	Dynein regulatory complex protein 1	3.7976	0.00015676
c80202_g3	Cyclin-dependent kinase-like 1	5.1672	3.84 × 10^-6^


*^∗^FDR: False discovery rate, which was used to determine the *p*-value threshold in multiple tests.*

### Analysis of Functional Enrichment

**FIGURE 2:**
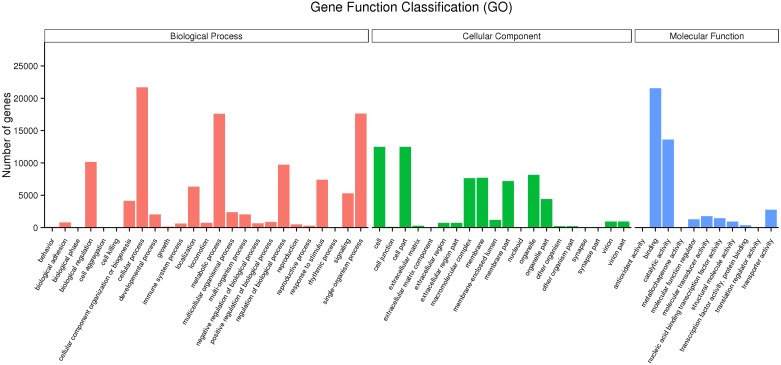
Gene ontology (GO) classification of the unigenes.

Mapping all the DEGs to terms in the GO database enabled the annotation of 149,862 unigenes, of which 65,580, 43,934, and 111,357 unigenes could be grouped into the cellular component, molecular function and biological process categories, respectively. In the cellular component category, cell (12,494, 32.76%), cell part (12,494, 32.76%), organelle (8,160, 21.39%), membrane (7,697, 20.18%), and macromolecular complex (7,636, 20.02%) represented the majority. Binding (21,572, 56.56%), catalytic activity (13,639, 35.76%), transporter activity (2,760, 7.24%), molecular transducer activity (1,776, 4.65%) and nucleic acid binding transcription factor activity (1,464, 3.84%) showed a higher proportion in the classification of molecular functions. Additionally, cellular process (21,702, 56.90%), single-organism process (17,647, 46.27%), metabolic process (17,595, 46.13%), biological regulation (10,164, 26.64%) regulation of biological process (9,741, 25.54%) and localization (6,333, 16.61%) represented the majority of the biological process categories (Figure 2).

**FIGURE 3:**
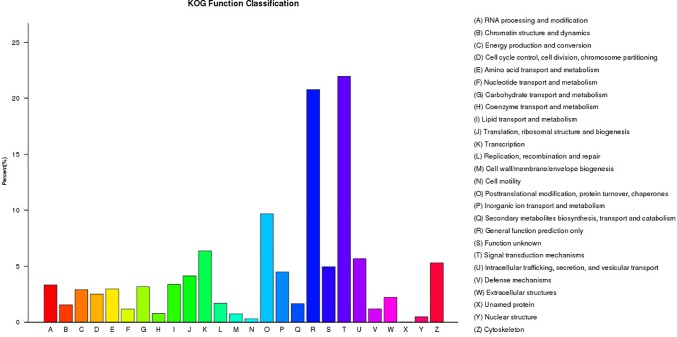
The KOG classifications of the unigenes.

A total of 21,304 unigenes were assigned to COG classifications (Figure 3). Among the 24 KOG categories, the top 10 categories were as follows: (1) signal transduction mechanisms (4,678, 21.96%), (2) general function prediction (4,427, 20.18%), (3) post-translational modification, protein turn-over, and chaperones (2,064, 9.69%), (4) transcription (1,355, 6.36%), (5) intracellular trafficking, secretion, and vesicular transport (1,210, 5.68%), (6) cytoskeleton (1,129, 5.30%), (7) function unknown (1,054, 4.95%), (8) inorganic ion transport and metabolism (955, 4.48%), (9) translation, ribosomal structure and biogenesis (882, 4.14%) and (10) lipid transport and metabolism (719, 3.37%). KEGG pathway annotation enabled us to assign the 10,023 DEGs to 232 pathways. In the enrichment analysis, the first ten enriched pathways included Ribosome (Ko1400), Fatty acid metabolism (Ko8746), Cell division (Ko04110), Oocyte meiosis (Ko04114), p53 signaling pathway (Ko04115), Focal adhesion (Ko04510), Adherens junction (Ko04520), Signaling pathways regulating pluripotency of stem cells (Ko04550), Regulation of autophagy (Ko04140) and Lysosome (Ko04142). These enriched pathways had functions in cell proliferation, steroidogenesis activity, receptor binding, and energy metabolism, which might indicate the differences in the developmental process of ovarian tissues between RCC and 4nRR fish.

Using log ratio values, we performed hierarchical clustering of 16,581 DEGs based on their expression. Expression levels during the stages of ovarian development were divided into 24 categories based on K-means clustering. Detailed expression profile clusters between 4nRR and RCC fish are shown in Supplementary Figure 4. The expression patterns not only indicate the diverse and complex interactions among genes, but also suggest that unigenes with similar expression patterns may have similar functions in the development of ovary.

### Validation of Differentially Expressed Genes by qPCR

**FIGURE 4:**
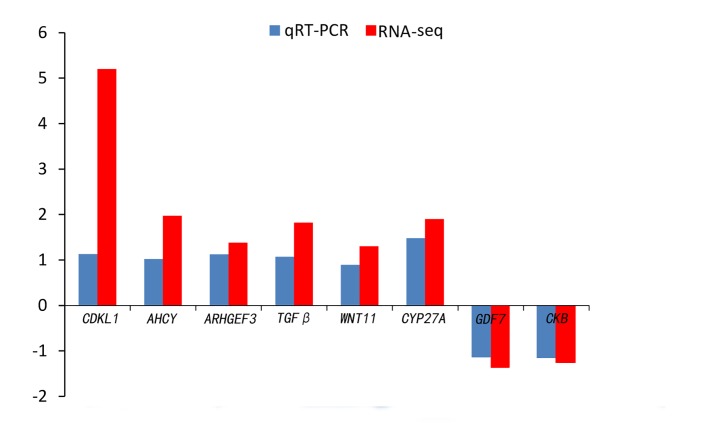
Comparison of the expression level of RNA-seq with qRT-PCR results between 4nRR and RCC fish.

**FIGURE 5:**
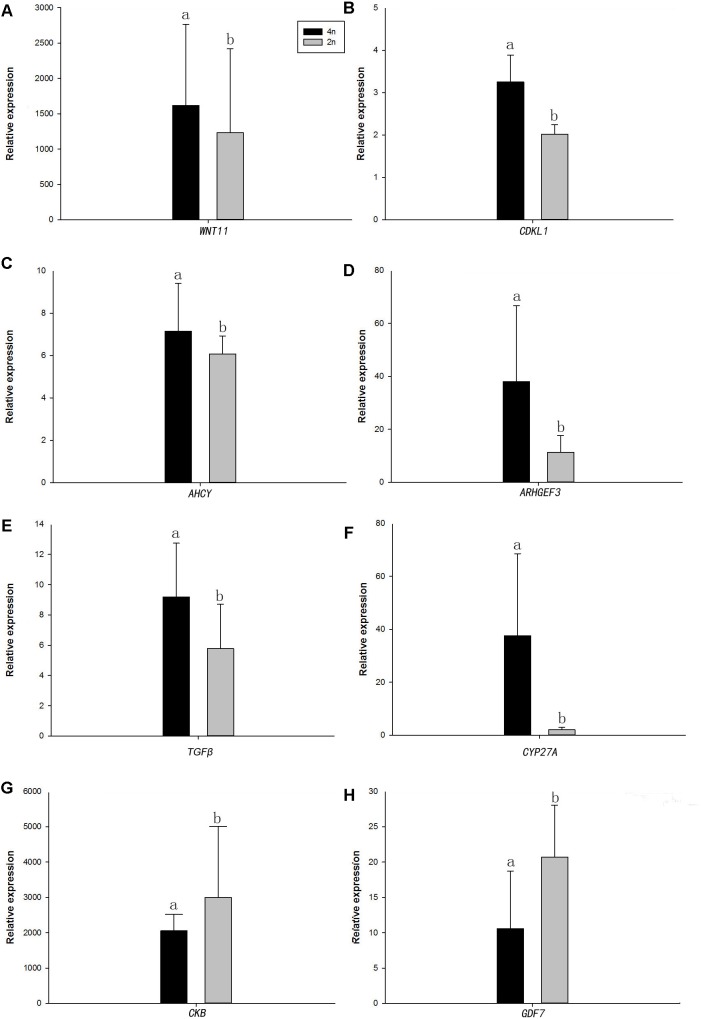
Real-time PCR analysis of the eight DEGs. **(A)** *WNT11*, protein Wnt11; **(B)** *CDKL1*, Creatine kinase B-type; **(C)** *AHCY*, adenosylhomocysteinase; **(D)** *ARHGEF3*, Rho guanine nucleotide exchange factor (GEF)3; **(E)** *TGFβ*, transforming growth factor beta; **(F)** *CYP27A*, vitamin D3-25 hydroxylase; **(G)** *CKB*, Creatine kinase B-type; and **(H)** *GDF7*, growth/differentiation factor 7. In each panel, 4n means 4nRR fish and 2n means RCC fish. Different lowercase letters indicate significant differences (*p* < 0.05) (means ± SD of relative expression; *n* = 9 for each group).

Quantitative real-time PCR was performed on 8 selected genes (*cyclin-dependent kinase-like* [*CDKL1*], *Creatine kinase B-type* [*CKB*], *adenosylhomocysteinase* [*AHCY*], *Rho guanine nucleotide exchange factor* (*GEF*)*3* [*ARHGEF3*], *transforming growth factor beta* [*TGFβ*], *growth/differentiation factor 7* [*GDF7*], *protein Wnt-11* [*WNT11*], and *vitamin D3-25 hydroxylase* [*CYP27A*]). The qPCR results were compared with the RNA-seq expression profiles (Supplementary Tables 3, 4 and Figure 4). The expression patterns of the eight genes by qPCR ranged from significantly different to similar to those indicated by the RNA-seq analysis (Figure 5).

## Discussion

### Significance of Polyploidization

Polyploidization of chromosomes was thought to be one of the most important mechanisms in species evolution (Masterson, 1994). Polyploidization is a major factor that drives plant genome evolution (Stupar et al., 2007) and fish evolution (Finn and Kristoffersen, 2007). Polyploidization not only significantly shaped the genomes but also affected other genetic aspects including gene expression (Cheung et al., 2009). Polyploids may contain genomes from different parental species (allopolyploidy) (Wang et al., 2017) or multiple sets of the same genome (autopolyploidy). Many studies have revealed that polyploid genomes undergo major chromosomal, genomic, and genetic changes (Doyle et al., 2008; Buggs et al., 2011, 2012; Ainouche et al., 2012). Despite the great progress in clarifying the genomic and transcriptomic changes that accompany polyploidization, few studies have explicitly correlated these changes with phenotype alterations (Gaeta et al., 2007). The changes in the characteristics of polyploids were mainly caused by differences in gene expression (Stupar et al., 2007; Chen et al., 2010), and thus, RNA-seq technologies can now be used in a high-throughput manner to investigate such phenotypic changes (Cui et al., 2013; Qiao et al., 2013; Zhang et al., 2017). Here, we showed that autotetraploidization causes increased egg size in 4nRR fish compared to RCC fish. We established a 4nRR fish lineage to better understand the genetic impact imposed by autopolyploidization. The 4nRR fish were derived from a whole genome duplication of RCC and possessed four sets of chromosomes derived from RCC (Qin et al., 2014). However, phenotypic changes were present in the 4nRR fish, including increased blood cell and germ cell sizes compared with RCC fish. Notably, the phenotypic and molecular data reported here were due to autopolyploidy rather than cultivar influence, as similar effects on the RCC and 4nRR fish cultivars were found.

### Significance of Egg Size Study

Autopolyploidy is traditionally considered to cause reduce fertility or sterility compared with diploid progenitors (Cifuentes et al., 2013). However, recent research showed that 4nRR fish can produce unreduced diploid eggs and showed dual reproductive modes of sexual reproduction and gynogenesis (Qin et al., 2015). In this research, the histological features of the gonads revealed that the 4nRR and RCC fish both possessed normal gonadal structure and could reach maturation. In the breeding seasons, large numbers of eggs were harvested from one-year-old 4nRR and RCC fish. These results showed that autotetraploidization did not cause fertility or sterility. Previous studies suggested that polyploid formation could induce various types of genomic changes (Wang et al., 2017). Comparative analysis based on egg size measurements revealed that the average diameter of diploid eggs from 4nRR fish was 17.71 mm, which was significantly larger than average haploid eggs with a diameter of 13.67 mm in RCC, suggesting that genetic factors were likely to be the cause of this difference in ovary development and egg diameter. In mature ovaries, the increased oocyte volume was mainly due to the incorporation of vitellogenin (Santos et al., 2007; Schilling et al., 2015). This process requires a range of enzymes to provide hormonal and energy support for the synthesis and breakdown of vitellogenin (Williams et al., 2014). We found that the egg diameters of 4nRR fish were obviously larger than those of RCC fish. Developing oocytes were thought to be largely non-transcribed and serve as a repository for specific maternal RNA, proteins and other molecules important for fertilization, initiation of zygotic development, and transition to embryonic gene expression (Santos et al., 2007; Reading et al., 2013; Chapman et al., 2014). Through self-mating experiments between 4nRR and RCC fish, we found that the fertilization of 4nRR fish to be lower than that of RCC. The result showed that variation in sizes of fish eggs has been associated with polyploidization. Among the 19,051 DEGs identified in this study, most of the key genes were involved in protein processing, fat and energy metabolism, cytoskeleton, steroidogenesis activities and cell division.

Cluster analysis of the genes differentially expressed between 4nRR and RCC fish identified a list of genes, of which 12,591 were more highly expressed in 4nRR fish and 6,424 were more highly expressed in RCC fish. With reference to the relevant literature (Heringstad et al., 2000; Dubrac et al., 2005; Menges et al., 2005; Knoll-Gellida et al., 2006; Santos et al., 2007), we screened 8 key genes (*CDKL1*, *AHCY*, *ARHGEF3*, *TGFβ*, *WNT11*, *CYP27A*, *GDF7* and *CKB*) related to egg development. Compared with RCC, there existed some genes in 4nRR fish that showed a marked up-regulation, (specifically *CDKL1*, *AHCY*, *ARHGEF3*, *TGFβ*, *WNT11*, *CYP27A*, *GDF7* and *CKB*) which might account for the differences in the egg diameters between RCC and 4nRR fish. *CDKL1* was a member of the *cyclin-dependent kinase-like* (*CDK*) protein family, which was a group of serine/threonine kinases (Santos et al., 2007). The cyclin dependent kinase *CDKL1* controls the cell cycle, which was best understood in the model organism *Saccharomyces cerevisiae. AHCY* (S-adenosylhomocysteine hydrolase) was the cellular enzyme that cells rely on for replication (Heringstad et al., 2000). *ARHGEF3* was a regulatory small GTPase that mediates signal transduction (Mullin et al., 2008) and was related to energy metabolism. *Transforming growth factor β* (*TGFβ*) and its signaling effectors act as key determinants of carcinoma cell behaviors, which play a key role in steroid hormone and vitellogenin synthesis during ovary development (Knoll-Gellida et al., 2006). *WNT11* regulates cell fate and patterns during embryogenesis. In many different tissues, *CYP27A* played an important role in cholesterol and bile acid metabolism and fatty acid metabolism (Dubrac et al., 2005). In our previous study, obvious expression difference of the *gnrh2*, *gthb* and *gthr* were found in the 4nRR fish (Qin et al., 2018). Altogether, our results provide a foundation for the further characterization of gene expression in 4nRR and RCC fish with respect to egg size.

## Data Availability

The datasets generated for this study can be found in National Center for Biotechnology Information, SAMN07418623 and SAMN07418624.

## Author Contributions

SL and QQ conceived and designed the study. YW and YP contributed to the experimental work and wrote the manuscript. MZ, XH, and LC performed most of the statistical analyses. YW and WL designed the primers and performed the bioinformatics analyses. MT and CZ collected the photographs. All authors read and approved the final manuscript.

## Conflict of Interest Statement

The authors declare that the research was conducted in the absence of any commercial or financial relationships that could be construed as a potential conflict of interest.

## Footnotes

1 https://www.ncbi.nlm.nih.gov/biosample?Db=biosample&DbFrom=bioproject&Cmd=Link&LinkName=bioproject_biosample&LinkReadableName=BioSample&ordinalpos=1&IdsFromResult=395975
