# Supplementary material for: Transcriptome Profile Analysis on Ovarian Tissues of Autotetraploid Fish and Diploid Red Crucian Carp
Source: Front Genet. 2019 Mar 19;10:208. doi: 10.3389/fgene.2019.00208 (PMC6434244; doi:10.3389/fgene.2019.00208)
Supplement: Supplementary file 1 [file Table_1.doc]

**Supplementary Figure legends**

**Supplementary Figure 1** The layout of the study design

**
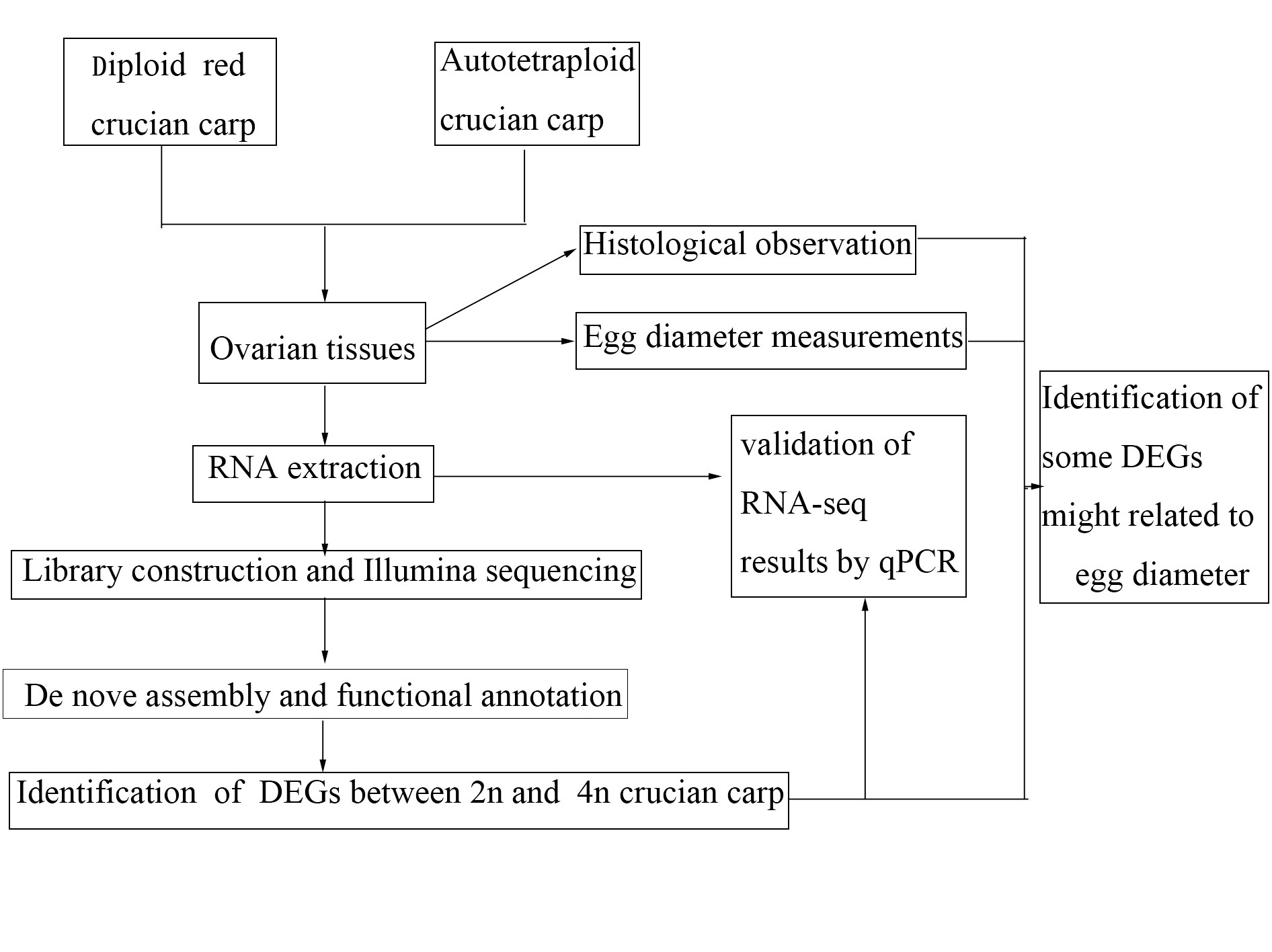
**

**Supplementary Figure 2** Length distribution of the transcripts and unigenes

**
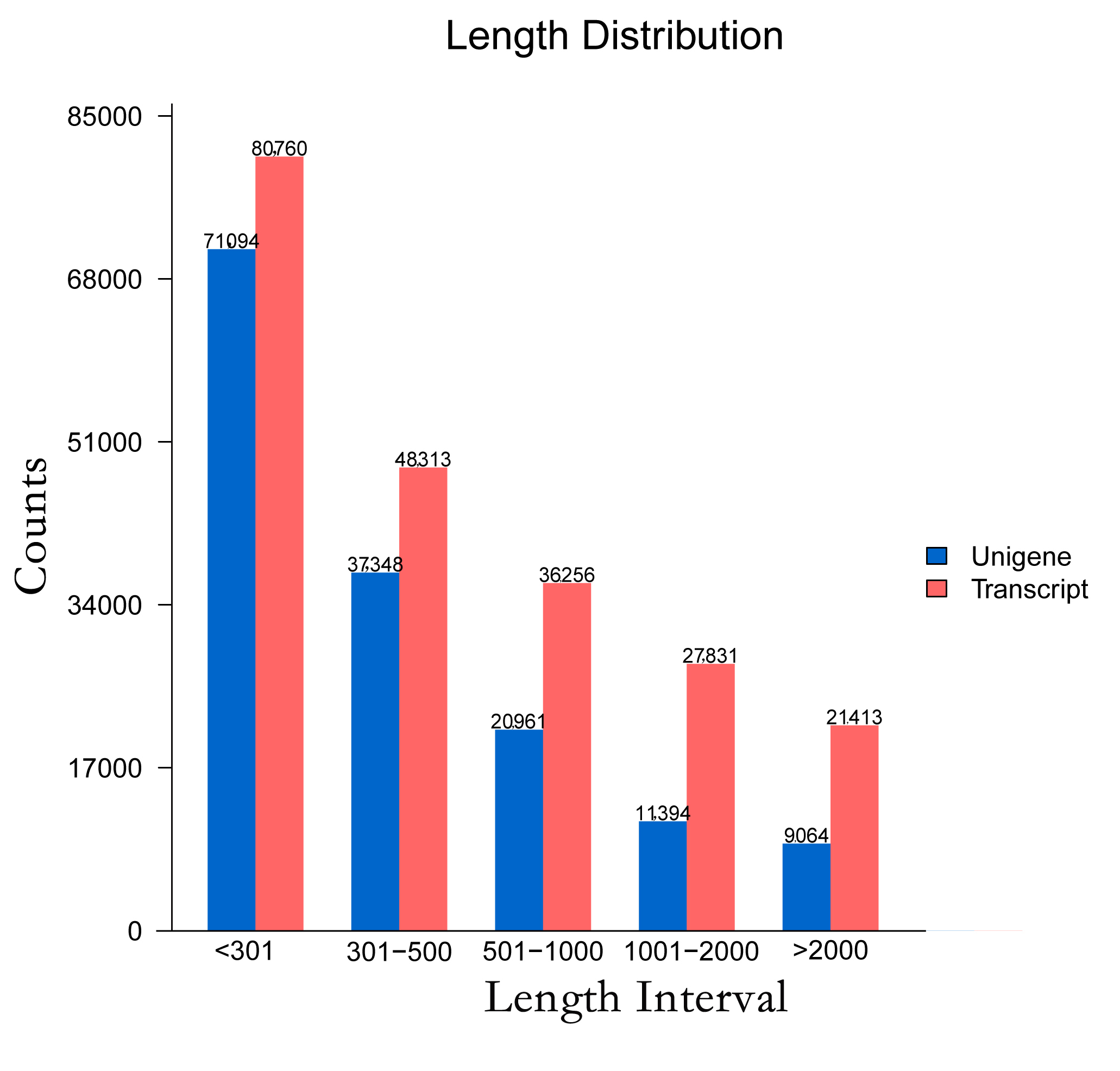
**

**Supplementary Figure 3** Unigenes were annotated using seven public databases (GO, KO, KOG, Nr, Nt, Pfam and SwissProt)

**
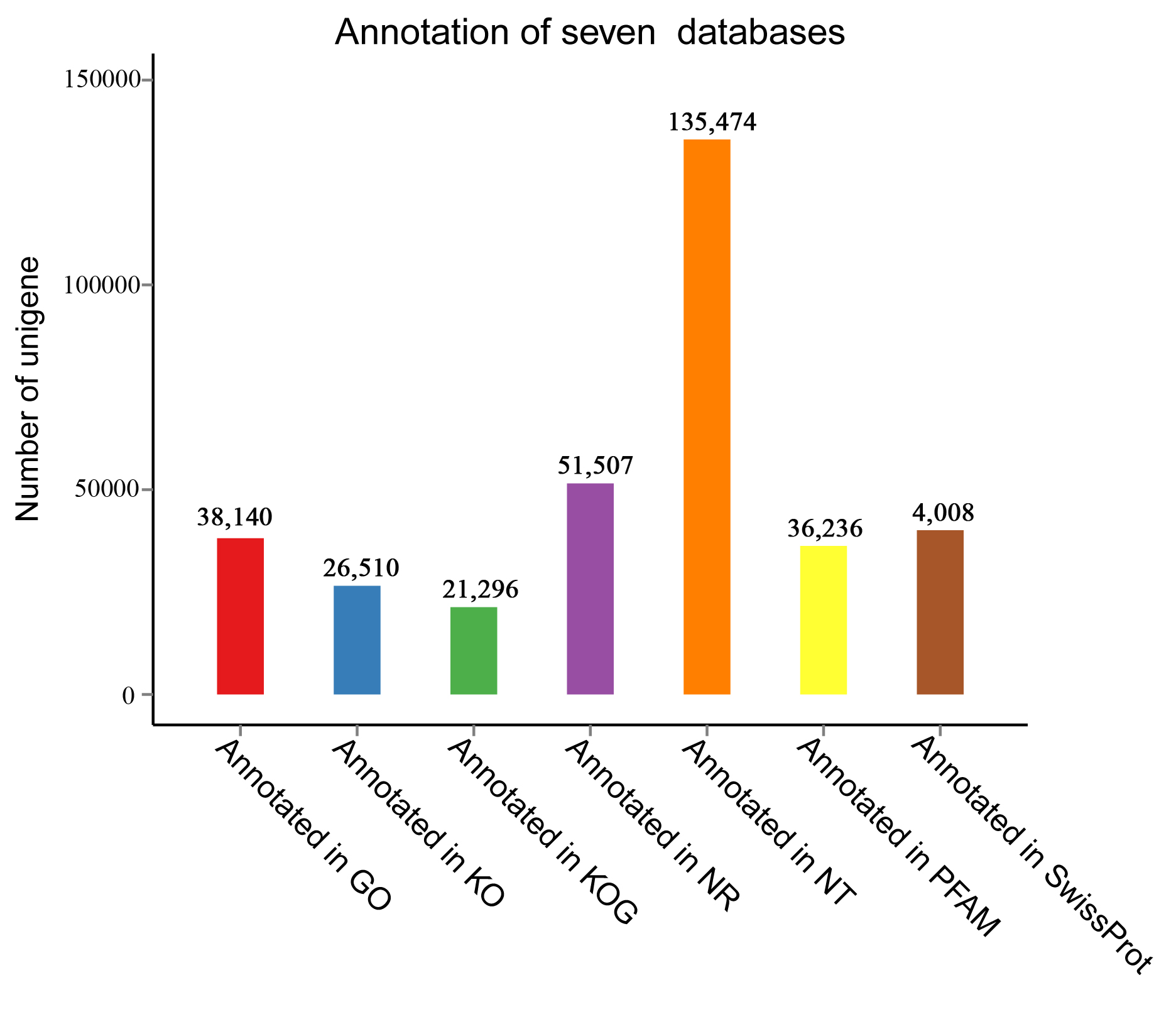
**

**Supplementary Figure 4** Hierarchical clustering of 16,581 unigene. Red represents upregulation and blue represents downregulation.

**
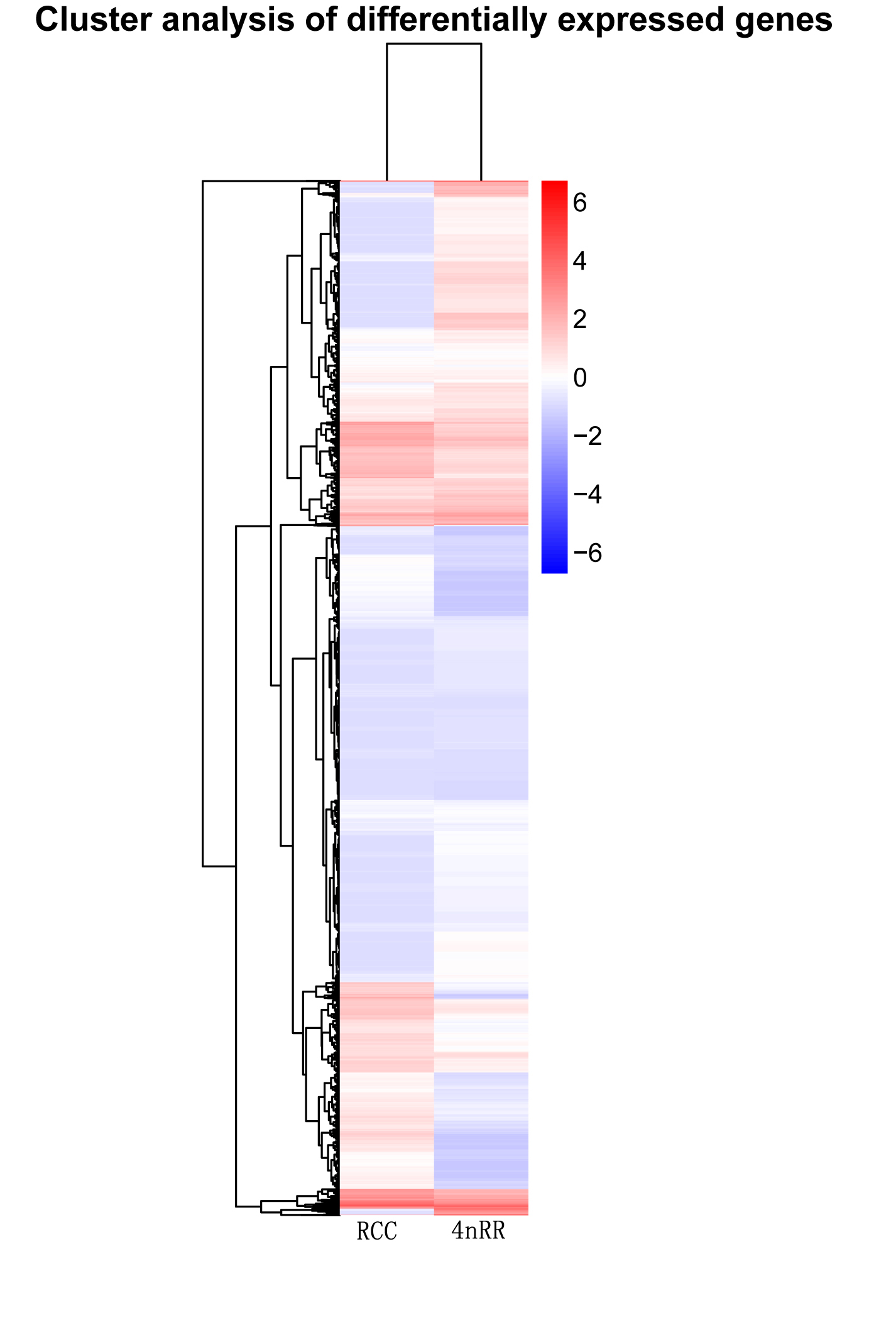
**

**Supplementary Table legends**

**Supplementary Table 1** Summary of Illumina expressed short reads production and filtering.

| Parameters | Red Crucian Carp | Autotetraploid Fish | In total |
| --- | --- | --- | --- |
| Number of raw reads | 63,416,982 | 54,714,586 | 118,131,568 |
| Average length of Raw read(bp) | 150 | 150 | 150 |
| Number of clean reads | 58,499,924 | 49,640,444 | 108,140,368 |
| Percentage retained | 92.24% | 90.72% | 91.54% |
| Average clean read length(bp) | 150 | 150 | 150 |

**Supplementary Table 2** Summary of the assembly.

| Length Range | Transcripts | Unigenes |
| --- | --- | --- |
| 200-500 | 129073 | 108442 |
| 500-1000 | 36256 | 20961 |
| 1000-2000 | 27831 | 11394 |
| >2000 | 21413 | 9064 |
| Total number | 214573 | 149861 |
| Mean Length | 380 | 311 |
| N50 length | 1525 | 996 |

**Supplementary Table 3** Primers used for quantitative real-time PCR (qPCR) verification.

| Gene-ID | Nr_annotation | Forward primer (5′-3′) | Reverse Primer (5′-3′) product size |
| --- | --- | --- | --- |
| c80202_g3 | *CDKL1* | ACGAGAACTTTGAGCGAGATG | CTCAGCCACTATTAGGGTCTTTAC 120 |
| C87059_g1 | *AHCY* | CTTGCACCTACCCAATTTCGA | TCACTGGTTAATGCAGGTCTC 115 |
| C96438_g1 | *ARHGEF3* | TCTCAGACTCCGAGGGAATGGT | CGGTTGGCAGAAGTTATGGGT 123 |
| C96591_g1 | *TGFβ* | GTTACAACCCAGAGCGAGAC | CGTGAGTGGTCCAGTATAAATC 108 |
| C92637_g1 | *WNT11* | CAGGACCACAGGCATTTAGAC | AGCCAGAAACACCGTGACATT 126 |
| C80533_g1 | *CYP27A* | TGCTCGTGTCGTTGCTGAAAG | AGGGAGGAAGGCAGATGGAT 145 |
| C80635_g1 | *GDF7* | TTGTTGCGTTCCTACTAAGCTC | CCTCACTCACATTGCTACCTG 132 |
| C77068_g2 | *CKB* | TGCCTAATCTCAGCAAATACCG | TTGACTCCATCCACCACCATC 125 |

**Supplementary Table 4** qPCR validation of randomly selected genes.

| Item | Up-regulated genes | | | | | | Down-regulated genes | |
| --- | --- | --- | --- | --- | --- | --- | --- | --- |
| Gene | *CDKL1*  5.2 | *AHCY* | *ARHGEF3* | *TGFβ* | *WNT11* | *CYP27A* | *GDF7* | *CKB* |
| RNA-seq |  | 1.97 | 1.38 | 1.82 | 1.30 | 1.90 | -1.37 | -1.26 |
